# Folk beliefs of cultural changes in China

**DOI:** 10.3389/fpsyg.2014.01066

**Published:** 2014-09-24

**Authors:** Yi Xu, Takeshi Hamamura

**Affiliations:** ^1^Department of Psychology, Chinese University of Hong KongHong Kong, China; ^2^School of Psychology and Speech Pathology, Curtin UniversityPerth, WA, Australia

**Keywords:** cultural change, modernization, folk beliefs, Ngram, cross-temporal analysis

## Abstract

For the last several decades, Chinese society has experienced transformative changes. How are these changes understood among Chinese people? To examine this question, Part 1 in this research solicited folk beliefs of cultural change from a group of Chinese participants in an open-ended format, and the generated folk beliefs were rated by another group of participants in Part 2 to gage each belief's level of agreement. Part 3 plotted the folk beliefs retained in Part 2 using the Google Ngram Viewer in order to infer the amount of intellectual interests that each belief has received cross-temporarily. These analyses suggested a few themes in Chinese folk beliefs of cultural change (1) rising perceived importance of materialism and individualism in understanding contemporary Chinese culture and Chinese psychology relative to those of the past (2) rising perceived importance of freedom, democracy and human rights and (3) enduring perceived importance of family relations and friendship as well as patriotism. Interestingly, findings from Parts 2 and 3 diverged somewhat, illuminating possible divergence between folk beliefs and intellectual interests especially for issues related to heritage of Confucianism.

## Introduction

During the last few decades, Chinese society has experienced a tremendous transformation driven by policy changes and rapid economic growth. The aim of the current research is to examine how Chinese people believe these changes have affected their culture and psychology. That is the current research investigates folk beliefs of cultural changes (Kashima et al., 2009) among Chinese.

In Kashima et al. (2009), folk beliefs of cultural changes among participants in Australia were found to resemble one influential theory of cultural change in social science—modernization theory—which postulates that societal changes triggered by economic growth induce systematic changes in cultural and psychological processes. Some evidence indicates that psychological impact of economic growth is understood in the similar way among Chinese participants (Cheng et al., 2010; Kashima et al., 2011). Based on this work, we anticipate that Chinese folk beliefs of cultural changes will also resemble the modernization theory.

In research, the modernization theory as a theory of cultural change has been challenged (Huntington, 1996; Kashima et al., 2009; Hamamura, 2012). In fact, some research has suggested that traditional cultural systems and their associated beliefs, values, and practices continue even in the face of societal modernization (Inglehart and Baker, 2000; Hamamura, 2012). Based on this work, we also anticipate that Chinese folk beliefs of cultural changes will reflect the theme of traditional cultural influence continuing.

The aim of this research is to gain an in-depth understanding of how these conflicting themes of cultural change—modernization effect and continuity of historical cultural influence—may shape contemporary Chinese folk beliefs of cultural changes.

Folk beliefs of cultural changes do not emerge in a vacuum. As such, the current investigation necessitates a review of available evidence on cross-temporal changes/continuity in Chinese culture and psychology. This review should enable a better understanding of the context within which folk beliefs of cultural changes are formulated. To organize this review, in the following sections we first present findings that are broadly consistent with the modernization theory. This is followed by a discussion of findings that are broadly inconsistent with the modernization theory and suggest cross-temporal continuity in Chinese culture and psychology.

### Research that suggests cross-temporal changes in chinese culture and psychology

The modernization theory has been very influential in shaping the conceptualization of cultural changes, not only within the community of researchers but also among lay people (Kashima et al., 2009, 2011; Cheng et al., 2010). The notion that the rapid modernization of Chinese society has made Chinese culture more individualistic is also prevalent in the literature.

In one study, Cai et al. (2012) examined whether socio-ecological factors that have become prevalent in recent decades in China—affluence, urbanization, and single-child households—are predictive of one psychological process sometimes associated with individualism, namely narcissism (Twenge et al., 2008). This research revealed that all three socio-ecological factors examined were independently predictive of narcissism—a higher score of narcissism was found among affluent (vs. less affluent) participants, those living in urban (vs. rural) areas, and those without (vs. with) siblings.

Evidence that converges with these patterns has been reported elsewhere. Hamamura et al. (2013) reported the effect of parental educational attainment on adolescents' tendency toward independence-interdependence. The effects of the one-child policy have also been examined extensively (e.g., Lee, 1992; Fan et al., 1994; Wang et al., 1998). One recent research compared the behaviors of participants from different birth cohorts using economic games and found that the cohorts born under the one-child policy were significantly less trusting, less trustworthy, more risk-averse, less competitive, more pessimistic, and less conscientious compared with those born before the introduction of the one-child policy (Cameron et al., 2013).

One specific change in Chinese culture that has been well documented is the psychological implications of shyness (Chen et al., 2005). Chen and colleagues have postulated that although shyness in Chinese culture had traditionally been a positively viewed trait seen as reflective of social maturity, shyness has been valued less in recent years, with a greater emphasis placed on competitiveness in socialization. To examine this idea, Chen and colleagues assessed three cohorts of 10-year-old children from Shanghai in 1990, 1998, and 2002. The study assessed the children's shyness and indices of their social functioning via peer assessment, teacher rating, and their academic performance. This research found that whereas shyness was positively associated with peer acceptance, teacher-rated competence, and academic achievement in the 1990 cohort, these associations were weaker in the 1998 cohort. Furthermore, shyness was negatively associated with peer acceptance and with teacher-rated school competence in the 2002 cohort. These findings suggest a cross-temporal shift in the psychological implication of shyness in Chinese socialization.

In sum, these findings suggest that changes in the socio-economic-political infrastructure in China have induced changes in Chinese psychology, such as a rising level of independence, pro-self orientation, and greater values placed on assertiveness in socialization.

### Research that suggests cross-temporal continuity in chinese culture and psychology

Not all available findings are consistent with the theme of rising individualism identified in the previous section. In fact, these findings suggest cross-temporal continuity in Chinese culture and psychology.

First, for the past few decades, research in cultural psychology has accumulated evidence indicating greater interdependence and collectivism among Chinese and other East Asians (Japanese and Koreans) relative to Westerners. This pattern has continued to emerge in recent years, for example, in comparisons between urban Chinese university students and American university students (Han and Northoff, 2008). These findings suggest that even after taking into account modernization in Chinese society, Chinese cultural and psychological processes continue to be relatively more interdependent and collectivistic.

In earlier work, these differences were articulated based on the considerations of differences in religious and intellectual traditions (e.g., Buddhism and Confucianism in China and Christianity and Greek philosophies in Western societies) (Markus and Kitayama, 1991; Nisbett et al., 2001). Recent work in this area has focused on ecological factors that have induced these cultural differences, differences in subsistence practices in particular.

Talhelm et al. (2014) have argued that a culture of interdependence emerges in regions that farm rice, whereas a culture of independence emerges in regions that farm wheat. The rationale for this theory is as follows. On the one hand, rice farming gives rise to an interdependent culture, as farming rice is highly labor intensive and also necessitates the coordination of labor to build and maintain an elaborate irrigation system and to plant and harvest rice within a short window of time. These labor practices gave rise to a culture that emphasizes cooperation and conflict avoidance. On the other hand, wheat farming does not require as elaborate a system of irrigation or as rigid a coordination of labor, giving rise to a culture of independence. Importantly, this theory postulates that the history of wheat/rice farming in a region exerts influence in shaping its contemporary culture and affects even non-farming residents, via a process akin to the survival of the culture of honor in the U.S. South (Nisbett and Cohen, 1996).

Talhelm and colleagues examined these predictions with a large number of Han Chinese students from six sites across China using multiple measures of independence-interdependence. The findings confirmed the pattern that participants from southern provinces, traditional rice farming regions, were more interdependent compared with those from northern provinces, traditional wheat farming regions. The implication of these findings is that the influence of historical subsistence practices—rice or wheat farming—continues to exert its influence in shaping modern Chinese culture and psychology.

In sum, findings from these studies suggest that the impact of historical socio-economic practices continues to exert its influence on contemporary Chinese culture and Chinese psychology.

### Current research

As mentioned earlier, the purpose of the literature review above was to depict the socio-historical context within which folk beliefs of cultural changes are formulated. The review suggests two themes of cross-temporal trends in Chinese culture and psychology: the rise of individualism (decline of collectivism) and the continuity of some traditional cultural influence.

The study reported below examined whether these two broad themes are evident in Chinese folk beliefs of cultural changes. In examining these possibilities, the current research takes a data-driven bottom-up approach, and inferences drawn this way are synthesized with the two broad themes discussed above as the framework.

The current research consists of three parts. Part 1 solicited folk beliefs of cultural changes on a wide variety of topics from a small group of Chinese participants in an open-ended format. These participants were asked to generate a list of topics and issues, in their opinion, that have become more or less important in understanding Chinese people of the past and present.

In Part 2, these responses were rated by a larger number of participants to gage each belief's prominence in Chinese folk beliefs of cultural changes—this procedure differentiated those folk beliefs that are more agreed upon from those that are more idiosyncratic. Based on the folk beliefs retained in this procedure, patterns in Chinese folk beliefs on cultural changes were inferred.

Part 3 of the study attempted to gage intellectual interest in the folk beliefs identified in Part 2 by analyzing them with the Google Ngram database. A detailed rationale for this analysis is discussed below, but in essence, the aim of Part 3 was to examine whether there is a correspondence between folk beliefs of cultural changes identified in Part 2 and intellectual interest in a given topic as inferred from the analyses of the Google Ngram database.

## Part 1. soliciting folk beliefs of cultural changes

We asked 11 students from Renmin University of China to generate a list of topics, either a single word or phrase, that they would associate as capturing similarities and differences between Chinese people of today and 50 years ago in an open-ended format.

To facilitate responding, the students were asked to list topics for the following domains: mental health/happiness, customs, values, relations with family and friends, relations with community and society, personality, and any other topics. The students were encouraged to write down as many topics as they could.

After screening for duplications and for non-sense responses, this procedure resulted in 74 unique responses. Note, although some of the responses shared a similar meaning, we decided not to remove such responses to guard against the possibility that topics with a seemingly similar meaning rated differently in Part 2.

## Part 2. gauging the degree of agreement among the generated folk beliefs

### Participants

We recruited 124 participants (46% female, average age 37.7) via an online company that hosts recruitment notices for surveys and that is used by millions of users in China (sojump.com). The participants were from different regions in China (e.g., Beijing 12%, Guangdong 19%, Shanghai 19%, Zhejiang 6%). All the participants had at least a high school education, with 69% with a college degree and 12% with a master's degree.

This sample is a biased sample of the Chinese population in at least a few ways, most notably in terms of high educational attainment. Arguably, the current sample (also the sample in Part 1) has a more articulated understanding of Chinese cultural changes. For this reason, the extent to which the inferences made below are applicable to a more representative sample of Chinese population is an important future question.

### Procedure

For each of the 74 topics from Part 1, the participants were asked about (1) its perceived importance in understanding similarities and differences between Chinese people of today and 50 years ago and (2) the perceived level of attention the topic has drawn in Chinese society.

For the perceived importance question, the participants were asked to select one of five options: (a) more important for understanding Chinese people of today, (b) more important for understanding Chinese people of the past, (c) always important, (d) never really important, and (e) don't know.

Similarly, for the perceived level of attention question, the participants selected one of five options: (a) more attention today than in the past, (b) more attention in the past than today, (c) always received much attention, (d) not much attention either past or present, and (e) don't know. These two questions resulted in similar ratings.

Next, we examined the level of agreement among the participants in rating particular topics. There were 20 topics that were rated by 50% or more of the participants as (a) for both questions. That is, these are topics generally seen as more important in understanding Chinese people of today and as drawing more attention in Chinese society today than in the past. We labeled these topics as “Rising” topics. Table 1 lists these topics.

**Table 1:**
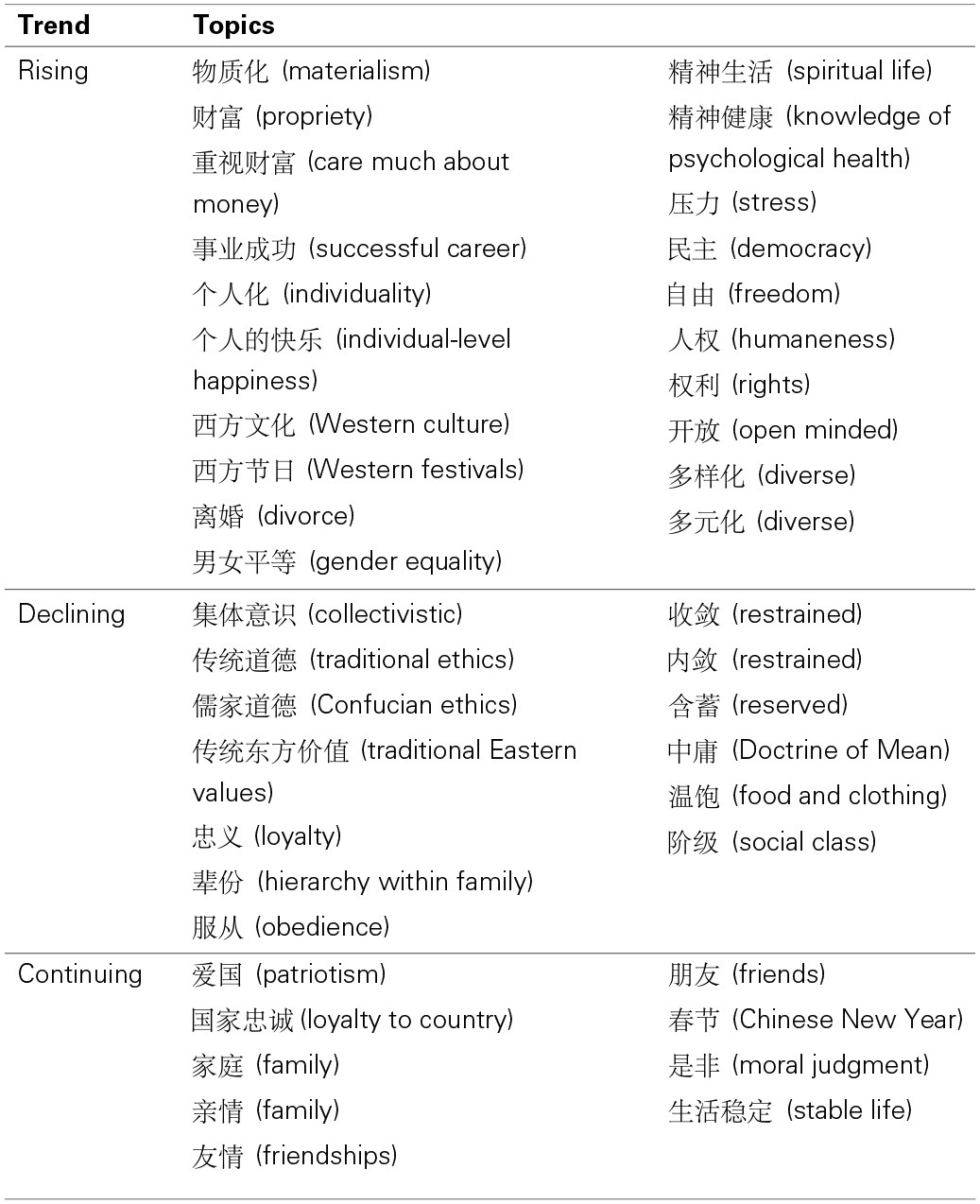
**Folk beliefs of cultural changes identified in Part 2**.

There were 13 topics that were rated by 40% or more^1^ of the participants as (b) for both questions. That is, these are topics generally seen as more important in understanding Chinese people of the past and as drawing less attention in Chinese society today than in the past. We labeled these topics as “Declining” topics.

There were nine topics that were rated by 40% or more of the participants as (c) for both questions. That is, these are topics generally seen as always important in understanding Chinese people and as always drawing much attention in Chinese society. These topics were labeled as “Continuing.”

#### Results

Our analysis focused on identifying patterns and commonalities among Rising, Declining, and Continuing topics.

***Rising topics***. Inspections of Rising topics suggest a few themes. The first theme is the perceived rising importance of wealth and materialism and related topics in understanding Chinese people of today than in the past. This theme was inferred from the perceived rising importance of materialism (
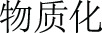
), wealth (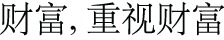), and career success (
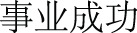
).

The second theme may be the rising perceived importance of individualism (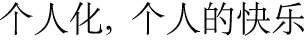) and Western cultural influence (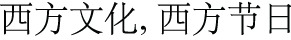). These trends may also capture the perceived rising importance of associated issues like divorce (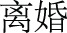) and gender equality (
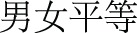
). In addition, this theme may also capture the perceived rising importance of psychological well-being (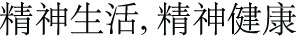). Alternatively, the perceived rising emphasis on psychological well-being may reflect the perceived rising importance of stress (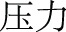).

The third theme may be the increased perceived importance of freedom (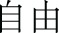) and democracy (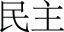) and human rights (
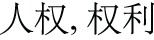
). In recent years, there has been an increasing knowledge and awareness of these issues among Chinese people stemming from their greater exposures to Western social practices. Our interpretation is that this trend may underlie the rising perceived importance of these issues.

Finally, the last theme may be the perceived rising importance of openness (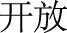) and acceptance of diversity (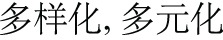). Our speculation is that these trends may reflect the impact of the transformative policy changes felt at the individual level. Although China under the Cold War was a largely secluded country, the country opened up with the introduction of the “open up and reform policy” in 1989, and the volume of economic and other forms of exchanges has steadily risen since then. This change might have induced greater exposures to and awareness of foreign cultures, as well as the means to afford foreign products with rising affluence (Vohra, 2000; Egri and Ralston, 2004). Our interpretation is that perceived increasing importance of openness and diversity in understanding Chinese people today may reflect this trend.

***Declining topics***. Inspections of the Declining topics also suggest a few themes. One theme is the opposite of rising individualism and Westernization—perceived declining importance of collectivistic and traditional ways of living. Topics relevant to this theme are collectivistic (
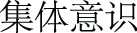
), traditional and Confucian ethics (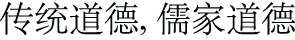), traditional Eastern values (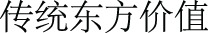), loyalty (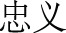), hierarchy within family (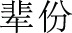), obedience (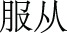), and Doctrine of Mean (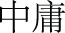)—from the teachings of Confucius. Topics associated with introversion, restrained, and reserved (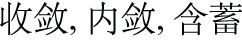) are also relevant to this theme because Confucianism emphasizes politeness and avoidance of causing offense or irritation to others, cultivating an introverted character, and having a reserved communication style (Yum, 1988; Gu, 1990; Yao, 2000).

Another trend may be the impact of historical events that have subsequently faded away, like the importance of food and clothing (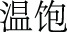), which was a salient societal issue (e.g., the Great Chinese Famine in 1960) but might have become less relevant with rising affluence. Similarly, the word “social class” (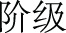) may carry specific connotations to the Cultural Revolution; hence, the declining importance of this topic that was perceived among the participants may reflect a declined influence of policies introduced during the Cultural Revolution. Note, although income disparity has become a societal issue in China in recent years, it is unlikely that the trend for social class (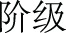) captures this trend, as the contemporary issue of growing gap between rich and poor is usually described by terms such as rich people (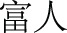) or powerful class (
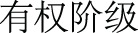
).

***Continuing topics***. Inspections of the Continuing topics suggest a theme that individuals' attachment to nation (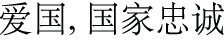), family (
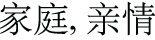
), and friends (
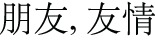
) is seen as enduring aspects of Chinese culture. Similarly, Chinese New Year (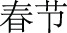) as the holiday for family reunions has been seen as an always important topic, as well as the desire for a stable life and harmonious family relationships (
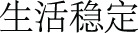
). Another theme may be the enduring perceived importance of moral judgment (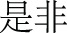).

## Part 3. plotting folk beliefs of cultural changes with the google ngram viewer

In Part 3, we plotted the folk beliefs identified in Part 2 in the Google Ngram database. The aim of this analysis was to examine whether there was a correspondence between a pattern found for a particular folk belief (i.e., rising, declining, continuing) and the amount of intellectual interest in that topic as inferred from analyses of the Chinese corpus of the Google Ngram database.

### Background on the google ngram viewer and its use in psychological research

The Google Ngram Viewer (https://books.google.com/ngrams/) plots the usage frequency of words/characters of interest based on Google's digitalized book archive, consisting of those books that were given to Google by partner publishers or from partner libraries. Google digitalized the contents of these books with optical character recognition technology and compiled them into a dataset that is searchable at the level of single words/characters, with the search results plotted by year.

For example, by entering “psychology” into the Google Ngram Viewer, it searches for all instances for which “psychology” is mentioned in the database, computes its occurrence for a particular year relative to a total number of all words that were used in all books published in that year that are archived in the database, and plots this percentage across years.

The Ngram Viewer is a valuable tool for conducting cultural product analysis (Morling and Lamoreaux, 2008), especially for investigating cultural changes. In fact, a few prior studies have incorporated analyses of the Ngram Viewer plots for this purpose. For example, Twenge and colleagues plotted the usage of words associated with individualism among contemporary Americans (e.g., “unique,” “all about me,” “I am the best”) and found that the usage of these words has increased in the American corpus since 1960 (Twenge et al., 2012). Greenfield (2013) analyzed the usages of words associated with psychological adaptation to urban environments (e.g., “choose” as opposed to “obliged” or “get” as opposed to “give”)—these words were chosen because “choose” is a defining attribute of individualism, while “obliged” is an important component of traditional collectivistic forms of living. Greenfield (2013) found increasing usages of these words in the American corpus between 1800 and 2000. (See also Oishi et al., 2013, for another usage of Ngram Viewer in inferring cultural changes).

As seen from these examples, the Ngram Viewer is a valuable tool for studying cultural changes. However, just as with any other research methods, analyses of Ngram Viewer plots have biases and errors that are currently inherent. Specifically, Michel et al. (2011) discussed issues of biased representations of books archived by Google and limitations of the technology involved in digitalizing books into a searchable database of words and characters.

The difficulty is compounded in analyzing the Chinese corpus for at least two reasons. First, currently, most of Google's partner publishers and libraries are based on Western countries. Hence, it's likely that a large percentage of the Chinese books digitalized in the database are those books that were originally acquired by Western libraries and were made available to Google through their partnership. As such, the database likely reflects a greater representation of those Chinese books that draw attention of Western audiences (e.g., the interests of Western academics in the case of Western university libraries). Hence, although the representativeness of the archive is also an issue for the English corpus (Michel et al., 2011), the issue is more problematic for the Chinese corpus.

The second issue is the censorship of book publication in China. For a book to be sold on the open market in China, it requires an approval from a government office, and books that are deemed inappropriate (e.g., those that touch on politically sensitive topics) are banned. In fact, Michel et al. (2011) demonstrated the effect of government censorship on the Chinese corpus. In particular, whereas the usage of the words “Tiananmen Square” was elevated in the English corpus following the Tiananmen Square protests in 1989, the corresponding pattern was not evident in the usage plot for its Chinese characters (
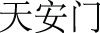
), suggesting a suppressed usage of these words in Chinese books. For this reason, Ngram plots for topics that are politically sensitive are unlikely to produce a pattern that genuinely reflects intellectual interest in those topics.

These issues necessitate caution from users of the Ngram Viewer for the Chinese corpus. Despite these issues, the Ngram database is arguably the richest source of data in inferring cultural changes that is currently available—its richness is revolutionary relative to other possible sources, which are usually very labor intensive (e.g., coding) and only contain data that is suitable for specific analyses (e.g., coded data of song lyrics for themes of individualism-collectivism (DeWall et al., 2011). For this reason, we believe that the current limitations of the Ngram Viewer plots for the Chinese corpus are best addressed by cautious interpretations of any resulting inferences and by acknowledging the tentative nature of any such interpretations. We present our findings below accordingly.

### Current analyses

The unit of analysis in the Google Ngram Viewer is n-gram, where 1-gram for Chinese is defined as a common semantic boundary (Michel et al., 2011). This means that a string of Chinese characters printed in a book that are scanned are segmented to many 1-grams based on their semantic boundary. As such, 1-gram in Chinese can consist of a single character (“
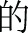
”) or multiple characters that from a semantic unit (two-character combinations such as “
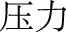
”). The Ngram Viewer also allows a search of phrases (i.e., two or more grams) by adding a space between 1-grams (e.g., a phrase “
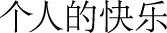
” was searched by adding a space between “
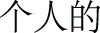
” and “
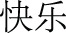
”).

In order for the Ngram Viewer plots to indicate reliable patterns, it is important that the target word appear with some frequency. For this reason, we did two things. First, we restricted our years of analysis to the period between 1980 and 2008 because the size of the Chinese corpus started to reliably exceed 10 million characters around 1980. 2008 is the last year for which the data is available currently. Second, we analyzed only those topics identified in Part 2 that had an average frequency of at least 1 in 1 million grams (or at least 0.0001%) since 1980 in the Ngram plots. With these two restrictions applied, all the patterns inferred below are based on topics that occurred at least 10 times in a given year in the Google Ngram database. Table 2 lists these topics, as well as their average usage frequency in the study period.

**Table 2:**
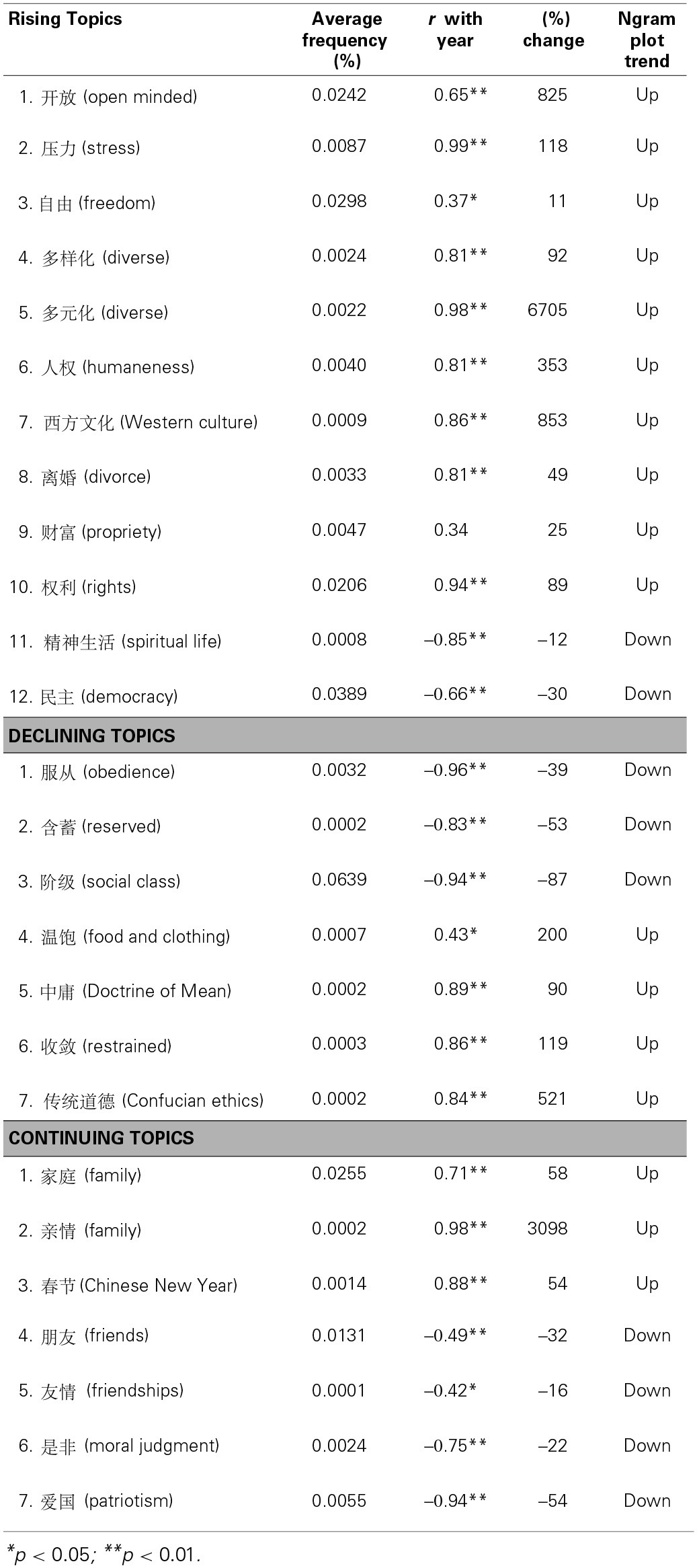
**Folk beliefs plotted in Part 3**.

^*^p < 0.05; ^**^p < 0.01.

In plotting figures and also for conducting all quantitative analyses, we used the “smoothing by 1” function. This means that a usage value for a given year was obtained by taking the average of a value for that year plus one value each for adjacent years on both sides. For example, the usage of “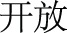 (open minded)” in 1990 accounted for 0.0254% of the entire corpus for that year. This value was summed with the corresponding value for 1989 (0.0257%) and 1991 (0.0288%), and the average of these three values (0.0266%) was the value for 1990. The value for the starting and ending years was obtained by averaging two values, the value for that year and the value for the single adjacent year (1980 and 2007, respectively, for the starting year and the ending year). We used this method to draw inferences that are close to the raw data yet are somewhat protected from outlying values.

## Results

For each topic that met the inclusion criteria above, we computed a correlation between its usage trend and year. Table 2 reports these correlations. To the extent that there is a correspondence between the folk beliefs identified in Part 2 and its usage trend plotted in the Ngram Viewer, this correlation should be positive for the Rising topics and negative for the Declining topics. We also analyzed the percentage changes in usage frequency between 1980 and 2008. On average, the topics analyzed indicated a large increase in usage frequency (Mean = 497%, Median = 56%, Min = −87%, Max = 6705%).

Because the analyses performed in the Ngram plots (e.g., correlations between frequency and year) are generally very sensitive to cross-temporal changes, the analyses for the Continuing topics are hard to interpret. As such, the analyses of these topics are reported in Table 2 but are not interpreted in detail.

### Rising topics

Among the 12 Rising topics, the correlation between usage frequency and year was positive for 10 topics (83% correspondence), with one correlation being marginal. The correlation was negative for two topics: democracy (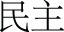) and spiritual life (
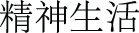
). That is, the usage frequency of these Rising topics actually indicated a downward trend.

**Figure 1:**
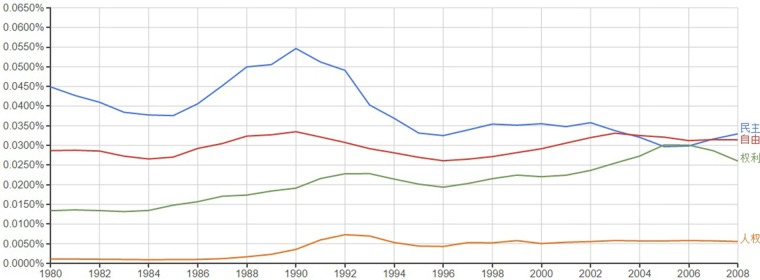
**An Ngram Viewer plot of freedom (**
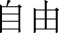
**.eps) and democracy (**
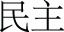
**.eps) and human rights (**
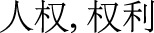
**.eps), from 1980 to 2008**.

A plot for democracy (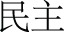) is presented in Figure 1. The plot shows a steep decline in the usage of this word in the early 1990s, the years following the Tiananmen Square protests of 1989. Hence, it may be that the declining trend reflects the suppression of intellectual interest in issues pertaining to democracy stemming from censorship. In Part 2, democracy was discussed along with freedom (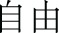) and human rights (
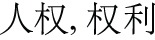
) as reflecting a rising awareness of these issues in Chinese society. Interestingly, the usage frequency of these words indicated rising trends corresponding to the rising pattern identified in Part 2.

As for the trend for spiritual life (
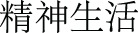
), although the Ngram plot indicates a declining usage of this word (*r* = −0.85), the magnitude of this change (−12%) is small—one of the smallest among the topics analyzed. Hence, an interpretation that we submit is that intellectual interest in spiritual life has been relatively unchanged.

### Declining topics

Among the seven Declining topics, the correlation between usage frequency and year was negative for three topics and positive for four topics. In terms of the correspondence between Parts 2 and 3, whereas the correspondence rate was 83% for Rising topics, it was only 43% for Declining topics.

Four Declining topics that indicated an upward trend in the Ngram plots were food and clothing (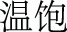), Doctrine of Mean (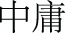), reserved (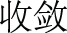), and traditional ethics (
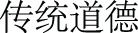
).

Regarding food and clothing (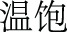), our interpretation is as follow: whereas issues of poverty and survival associated with China in earlier decades have largely been eradicated with rising affluence, the pace of economic development has been very uneven, leading to a new societal issue of a large inflow of population from rural areas into urban centers seeking a better standard of living. The upward trend of food and clothing (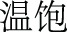) may capture intellectual interest in these issues. Alternatively, the upward pattern may reflect a rising intellectual interests for the traumatic issue that had affected livelihood of many that had been rectified in recent decades.

The plots for Doctrine of Mean (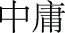), restrained (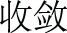), and traditional ethics (
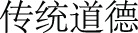
) showed an upward trend. As mentioned, these topics share in common their association with Confucianism. Although these topics were seen as declining in their importance in Part 2, patterns from the Ngram plots indicate rising intellectual interest in these topics. This pattern is discussed in detail in the General Discussion section.

## General discussion

This research examined folk beliefs of cultural changes among Chinese using a survey and the Google Ngram Viewer. Findings in Part 2 suggested a few trends in Chinese folk belief of cultural changes. First, some of the folk beliefs resembled tenets of the modernization theory, suggesting a greater perceived importance of materialism, individualism, and Westernization in understanding Chinese culture and Chinese psychology today. Conversely, analyses also suggested a declined perceived importance of traditional Chinese/Eastern cultural practices and collectivism in understanding Chinese culture and Chinese psychology.

In addition to this theme, the findings also suggested the perceived rising importance of freedom, democracy, and human rights in understanding contemporary Chinese culture and psychology. Our interpretation was that this trend may reflect the increased exposure to and greater awareness of Western social practices among Chinese, especially among the population sampled for the current study.

Finally, our analysis also identified a theme of cultural continuity within the Chinese folk beliefs with regards to the importance of family relations and friendships, as well as patriotism.

Part 3 cross-examined these trends using the Google Ngram Viewer. Analyses of the trends revealed a mixed pattern. On the one hand, for those folk beliefs that were seen as increasingly important in understanding Chinese in Part 2 (Rising topics), the Ngram Viewer plots tended to show a corresponding pattern—the rate of correspondence was 83%. On the other hand, the correspondence rate was much lower (43%) for those folk beliefs that were seen as declining in their importance in understanding Chinese (Declining topics).

Although not anticipated, analyses of these discrepancies afforded interesting insights. For example, the discrepancy identified for the word “democracy” (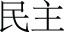)—Rising topic in Part 2 that showed a downward trend in the Ngram Viewer plot—seems to reflect the effect of government censorship of politically sensitive topics.

Among Declining topics in Part 2 that showed an upward trend in the Ngram Viewer plots, one theme we identified was rising intellectual interest in topics pertaining to Confucianism: Doctrine of Mean (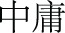), reserved (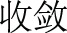), and traditional ethics (
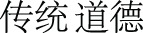
). Our interpretation of this pattern is that perceived declining importance of cultural traditions and intellectual heritage—as found in Part 2—has stirred intellectual interest in these issues. For example, although assertiveness in social interactions has become increasingly important in socialization (Chen et al., 2005), recent research continues to suggest that interpersonal relationships in China emphasize interpersonal harmony and conflict avoidance (Zhang et al., 2005). The increasing usage frequency of words associated with Confucianism identified in Part 3 may reflect a rising intellectual interest in these issues. The increasing usage frequency of words associated with family may also reflect the similar dynamics—it may be the case that traditional values surrounding family life are seen as eroding and have stirred intellectual interests in response.

In the current research, we interpreted Ngram Viewer plots as reflecting intellectual interests that a given topic has received. As such, one way to frame the discrepancies between the findings from Parts 2 and 3 may be that they reflect differences of folk beliefs of cultural change held among lay individuals and intellectuals. Another plausible interpretation would be that Ngram Viewer plots capture actual patterns of communication within society. Such an interpretation would have to assume some degrees of correspondence between the frequency in which a given topic is communicated within society and the frequency in which that topic is mentioned in book publications. Nevertheless, when interpreted this way, the current findings beg the question of why perceptions of cultural changes assessed directly (i.e., Part 2) sometimes diverged from the ways in which those topics are talked about in the society, as inferred from Ngram Viewer plots. Answer to this question may allude to the issue of inaccuracy in retrospectively judging historical patterns.

These interpretations, along with the limitations of the current study discussed earlier—issues of sample representativeness in Part 2 and potential errors and biases inherent in analyses of the Chinese corpus of the Google Ngram database—await further considerations in future research.

### Conflict of interest statement

The authors declare that the research was conducted in the absence of any commercial or financial relationships that could be construed as a potential conflict of interest.

## Footnotes

^1^For Declining and Continuing topics, the criteria for consensus was lower than Rising topic because the level of agreement among the raters was generally lower for Declining and Continuing topics than Rising topics. In fact, if the criteria for consensus were set at 50%, we would have only retained 3 out of 13 Declining topics reported here.
